# Characterization of a Self-renewing and Multi-potent Cell Population Isolated from Human Minor Salivary Glands

**DOI:** 10.1038/srep10106

**Published:** 2015-06-09

**Authors:** Lin Lu, Yan Li, Ming-juan Du, Chen Zhang, Xiang-yu Zhang, Hai-zhou Tong, Lei Liu, Ting-lu Han, Wan-di Li, Li Yan, Ning-bei Yin, Hai-dong Li, Zhen-min Zhao

**Affiliations:** 1 Research Center, Plastic Surgery Hospital, Chinese Academy of Medical Sciences and Peking Union Medical College, Beijing, PR China; 2 People’s Hospital of Jincheng City, Jincheng, Shanxi, PR China; 3 International Medical Plastic and Cosmetic Centre, China Meitan General Hospital, Beijing, PR China; 4 Department of Cosmetic and Plastic Surgery, Evercare Beijing Medical & Beauty Hospital, Beijing, PR China; 5 Microinvasive Department of Plastic Surgery, Plastic Surgery Hospital, Chinese Academy of Medical Sciences and Peking Union Medical College, 33 Ba Da Chu Road, Beijing, PR China; 6 Department of Cleft Lip and Palate, Plastic Surgery Hospital, Chinese Academy of Medical Sciences and Peking Union Medical College, 33 Ba Da Chu Road, Beijing, PR China

## Abstract

Adult stem cells play an important role in maintaining tissue homeostasis. Although these cells are found in many tissues, the presence of stem cells in the human minor salivary glands is not well explored. Using the explant culture method, we isolated a population of cells with self-renewal and differentiation capacities harboring that reside in the human minor salivary glands, called human minor salivary gland mesenchymal stem cells (hMSGMSCs). These cells show embryonic stem cell and mesenchymal stem cell phenotypes. Our results demonstrate that hMSGMSCs have the potential to undergo mesodermal, ectodermal and endodermal differentiation in conditioned culture systems *in vitro.* Furthermore*, in vivo* transplantation of hMSGMSCs into SCID mice after partial hepatectomy shows that hMSGMSCs are able to survive and engraft, characterized by the survival of labeled cells and the expression of the hepatocyte markers AFP and KRT18. These data demonstrate the existence of hMSGMSCs and suggest their potential in cell therapy and regenerative medicine.

Cell therapy offers exceptional opportunities for the treatment of human diseases and disabilities^1^, obviating the shortage of donor organs and the requirement for long-term immunosuppressive treatment^2^. Diseases targeted by cell therapy include vascular, neurological, autoimmune, liver and cardiovascular diseases^2^. So far, stem, progenitor, primary and genetically modified cells have been delivered via cell therapy. Among all the cell types, stem cells are the most favorable source due to their self-renewal and differentiation capacities^3^. Embryonic stem cells (ESCs) are derived from the inner cell mass of mammalian blastocysts, are self-renewing and can differentiate into any body-cell types^4^, however, the underlying risk of teratoma formation and immune rejection hinders their clinical application. Induced pluripotent stem cells (iPSCs) reprogrammed from somatic cells to produce pluripotent cells represent a promising autologous cell source except for its long-term genetic stability and tumorigenic potential. Adult stem cells are self-renewing cells present in adult tissues, and they can escape their quiescent state to maintain tissue homeostasis in the turnover process or in response to injury^5^. Adult stem cells serve as a more advantageous source in cell therapies, because they can be isolated from autologous tissues and are not tumorigenic when transplanted *in vivo*^2^; however, levels of engraftment and transdifferentiation detected within the injured tissue are low^6^. According to recent research, adult stem cells have been isolated from many tissue types, including bone marrow, adipose tissue, small intestine, mammary gland, breast milk and amniotic fluid^7^,^8^.

Oral tissue has been renowned for its regenerative capacity and quick healing with minimal post-biopsy scarring. Multi-potent stem cells have been found located in the lamina propria of the oral mucosa, exfoliated deciduous teeth and periodontal ligaments^9^,^10^,^11^,^12^. Salivary glands play a vital role in general well being as well as in oral health via the saliva. Human salivary gland system is composed of major and minor salivary glands. Major glands secrete saliva mainly upon stimulation, whereas minor glands continuously maintain the health of the oral cavity by saliva coating^13^,^14^. Minor salivary glands are located in the palatal, buccal, labial, and lingual parts of the mucosal membrane in the oral cavity^13^. Minor salivary glands biopsy is readily used for diagnostic purpose due to their accessibility in clinics^15^. Stem or progenitor cells have been isolated from major salivary glands, but there is limited knowledge about minor salivary glands^16^,^17^. In order to obtain stem cells from major salivary glands, a duct ligation model in mouse submandibular glands was used to induce regenerative progenitor cells, which implied cell differentiation into endodermal lineages^18^. Recently, an *in vivo* histone2B green fluorescent protein pulse-chase strategy has been used to define label-retaining cells in the mouse minor salivary glands^19^, but thus far, minimal data have described the presence of mesenchymal stem cells located in human minor salivary glands and little is known about their characteristics. Our group has been investigating human minor salivary glands, and we have previoiusly reported that cells isolated from minor salivary glands are capable of osteogenic differentiation^20^.

In this study, we report that culture of human minor salivary glands generates a novel mesenchymal stem cell population with self-renewal and multi-lineage differentiation capacities. We further provide detailed characterization of this population, namely, human minor salivary gland mesenchymal stem cells (hMSGMSCs). Moreover, we demonstrate for the first time that mesenchymal stem cells isolated from human minor salivary glands are able to generate cell repopulation in acute liver injury models, indicating potential multi-organ therapeutic application.

## Results

### Isolation and Proliferation Capacity of Cells Isolated from Human Minor Salivary Glands

**Figure 1:**
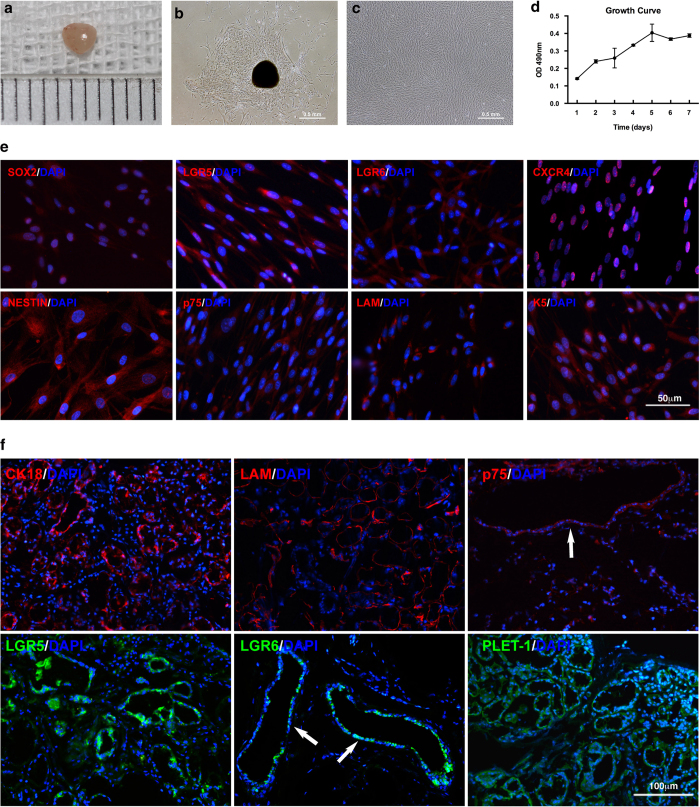
Culture of human minor salivary gland mesenchymal stem cells and expression of stem cell markers *in vitro* and *in vivo*. (**a**) One of the minor salivary glands dissected during surgery. (**b**) Explant primary culture for hMSGMSCs at day 4. (**c**) Expanded hMSGMSCs of passage 3. (d) Cell growth curve for passage 4 hMSGMSCs. (**e**) Immunostaining of ex vivo expanded hMSGMSCs. (**f**) Immunostaining of human minor salivary gland tissues for stem cell markers. Positively expressed stem cell markers in hMSGMSCs localize in the basal layer of lower secretory ducts (white arrows).

Surgically obtained human minor salivary glands were normally 2-4 mm in diameter (Fig. 1a). Surgical dissection and explant culture method were used to isolate the human minor salivary gland stem cells. Over 4-7 days, two different cell subpopulations migrated out the tissue (Fig. 1b): oval-shaped epithelial-like cells around the tissue explant and fusiform cells sparsely located outside the oval cells. The fusiform cells were isolated by the clone ring to obtain homogenous-shaped cells after expansion (Fig. 1c). Usually, each small salivary gland was able to yield 1 × 10^6^ cells after expansion at the first passage. Such cells were passaged at a ratio of 1:3 every 3-5 days. We were able to maintain the culture until passage 20 without obvious morphological changes. To determine the self-renewal ability of the cells isolated, colony formation and MTT proliferative assays were performed. The population doubling time was calculated as 66 hours using the MTT assay results, and a growth curve was also calculated (Fig. 1d). For cells at passage 4, the efficiency of colony formation was 41.00 ± 8.83%, which suggests the existence of a self-renewable population of human minor salivary gland mesenchymal stem cells (hMSGMSCs).

### Characterization of hMSGMSC Phenotypes

Flow cytometry analysis of the cell cultures at passage 3-5 (Table 1) shows that the most highly expressed cell surface markers were consistent with the profile of mesenchymal stem cells, including CD29, CD73, CD90, CD105 and CD166, with the exception of CD44, which has been suggested to be an epithelial marker^19^. The cells were shown to lack epithelial lineage-related markers such as CD49f, CD324 and CD338 (also known as ABCG-2). CD34 and CD45 were not detected, indicating non-hematopoietic origin. The expanded hMSGMSCs were >95% positive for human leukocyte antigen-ABC(HLA-ABC) and negative for human leukocyte antigen-DR(HLA-DR). The costimulatory surface antigens CD80 and CD86 were not expressed, suggesting the possibility of allogeneic utilization. These data indicate that these cells have immunological characteristics similar to those of human bone marrow-derived mesenchymal stem cells (hBMSCs). We then tested whether hMSGMSCs were positive for cell markers typical of hESCs^21^. Unlike most characterized mesenchymal stem cells, hMSGMSCs included a high percentage of SOX2-positive cells, as well as a population of NANOG- and SSEA-1- positive cells in hMSGMSCs^10^. Additionally, >95% of the expanded cells expressed nestin, which is a protein marker for neural stem cells^22^. These data suggested that hMSGMSCs share markers with other stem cells.

Next, we further confirmed the phenotypes by immunofluorescence in both expanded hMSGMSCs and human minor salivary gland tissues (Fig. 1e,f). The transcription factor SOX2 was specifically localized to the nuclei of hMSGMSCs. We also observed the expression of the G-protein coupled receptors LGR5 and LGR6, which are markers of intestine, liver^23^,^24^ and epithelial stem cells^25^. Nestin and p75, which are markers of neural crest stem cells, could also be detected in hMSGMSCs. Major proteins of the basal lamina, including laminin and KRT 5, were also positively expressed in hMSGMSCs. From the tissue histology staining, we were able to locate stem cell markers positive cells around the basal layer of the lower excretory duct. Taken together, these data indicated that hMSGMSCs showed phenotypes of mesenchymal stem cells, embryonic stem cells and other adult stem cells.

To show that hMSGMSCs could maintain the stem cell phenotypes in an *in vitro* culture system, we performed flow cytometry on cells cultured to passage 5,10,15 and 20. The cultured cells maintained high expression of CD29, CD44 and CD73 at each passage tested, whereas CD90, CD105 and SSEA-1 levels decreased during the course of culture (Supplemental Table 1). At passage 20, the majority of the cells in culture maintained expression of stem cell markers, which indicates that the phenotypes of hMSGMSCs can be maintained during culture *in vitro*.

### Differentiation Ability of hMSGMSCs *in Vitro*

To determine the differentiation potential of hMSGMSCs, we examined their lineage-directed differentiation in specific induction media, using methods previously reported for hESCs and hBMSCs.

#### Mesodermal differentiation of hMSGMSC

**Figure 2:**
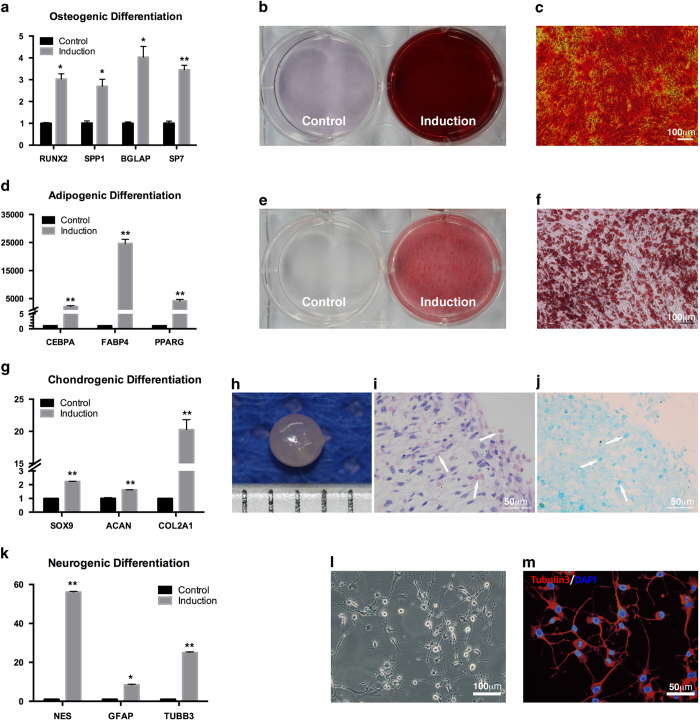
Multi-lineage differentiation of hMSGMSCs. Quantitative real-time PCR analysis of relative mRNA expression of markers for different lineages (**a**,**d**,**g**,**k**). Alizarin S red staining at day 21 of osteogenic induction, plate view (**b**), microscopic view (**c**). Oil red staining at day 14 of adipogenic induction, plate view (**e**), microscope view (**f**). Pellet formed at day 28 of chondrogenic induction (**h**). H&E staining (**i**) and Alician blue staining (**j**) of chondrogenic induced pellet, white arrow pointing to acidic polysaccharides in cartilage matrix. Cell morphology (**l**) and immunostaining for Tubulin 3 (**m**) post neurogenic induction for 5 hours are shown. *P < 0.05, **P < 0.01, n ≥ 3.

The potential for differentiation into the cell types of the mesoderm lineage was confirmed by directed osteogenic, adipogenic, and chondrogenic induction. After culture under osteogenic conditions for 14 days, we detected the significantly induction of the essential osteoblastic differentiation transcription factors *RUNX2* (P = 0.0165) and *SP7* (also known as osterix, P = 0.0036) as well as then mineralization markers *BGLAP* (also known as osteocalcin, P = 0.0198) and *SPP1* (also known as osteopontin, P = 0.0456) (Fig. 2a). Nodular mineralized deposits characteristic to osteoblasts were further identified using Alizarin Red S staining (Fig. 2b,c). For adipogenic lineage differentiation, significantly increased expression was observed for the adipogenic transcription factors *PPARG* (peroxisome proliferator-activated receptor gamma, P = 0.007) and *CEBPA* (also known as C/EBP-alpha, P = 0.0016) as well as the adipocyte marker FABP4 (also known as aP2, P = 0.0012), after 7 days of induction (Fig. 2d). To visualize the development of lipid droplets, fixed cells were stained with oil red. Large lipid droplets were clearly visible, suggesting adipogenesis from hMSGMSCs (Fig. 2e,f). Chondrogenic induction was performed using the pellet method. After induction for 14 days, cartilage-like pellets formed (Fig. 2h), and significant increases in the mRNA expression of the differentiation-related markers *SOX9* (P = 0.0052), *ACAN* (aggrecan, P = 0.0027), and *COL2A1* (collagen II, P = 0.0022) were detected with real-time PCR (Fig. 2g). The pellets were also analyzed using H&E, Alician blue staining, and immunohistological assays to identify collagen matrix development related to chondrocyte formation (Fig. 2i,j). In summary, we demonstrate that hMSGMSCs have the capacity to differentiate into mesoderm lineages under directed treatment.

#### Neuronal differentiation of hMSGMSCs

As shown above, hMSGMSCs are positive for p75 and nestin, as indicators of neural stem cells. We therefore further tested the potential for neural differentiation of hMSGMSCs. After induction for 5 hours, we observed a neural-cell morphology change (Fig. 2l). Additionally, we observed increased expression of *NES* (nestin, P = 0.0034), *GFAP* (glial fibrillary acidic protein, P = 0.0296) and *TUBB3* (tubulin beta 3 class III, P = 0.0089) at the mRNA level (Fig. 2k), with positive expression of Tubulin 3 protein after induction (Fig. 2m), indicating the neural differentiation potential of hMSGMSCs.

#### Endodermal differentiation of hMSGMSCs

**Figure 3:**
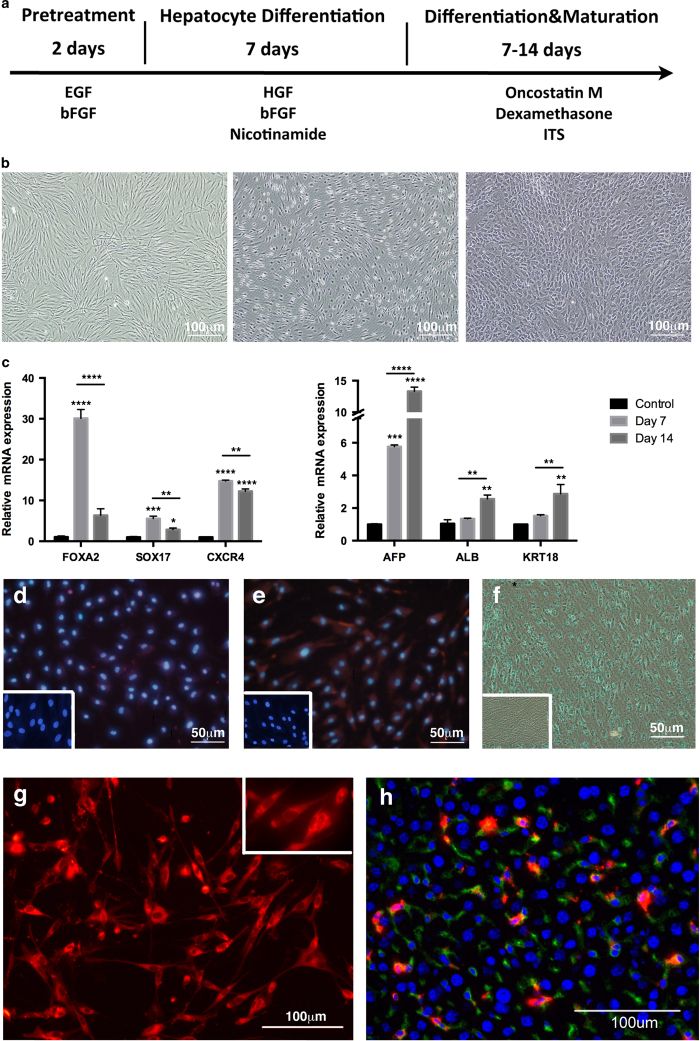
Hepatocyte induction *in vitro* and *in vivo*. Three-stage induction protocol for hepatocyte differentiation (**a**). Pictures taken on day 1, day 7 and day 14 from left to right(b). Quantitative real-time PCR analysis of relative mRNA expression of endoderm marker genes for different development process on day 7 and day 14 (**c**). Immunostaining for hepatocyte expressing protein AFP (**d**) and KRT 18 (**e**). Indocyanine green (ICG) assay on day 14 of hepatocyte induction (**f**). Cell image under fluorescence microscope 12 hours post CM-Dil labeling (**g**). AFP immunostaining 2 weeks after hMSGMSCs transplantation, green fluorescence signal for AFP and red for CM-Dil labeling(h). *P < 0.05, **P < 0.01, ***P < 0.001, **** P < 0.0001, n ≥ 3. Negative control images were show in the white rectangles in d, e and f.

To demonstrate that hMSGMSCs have the capacity to differentiate into endoderm lineages, we followed the three-stage hepatocyte induction protocol as described previously (Fig. 3a). Surprisingly, we observed an obvious morphological change from fibroblast-like into epidermis-like cells during the induction procedure (Fig. 3b). Intriguingly, consistent with the induction of the hepatocyte markers *AFP* (P < 0.0001), *ALB* (albumin, P = 0.0016) and *KRT18* (keratin 18, P = 0.0014) during the two stages, the endoderm lineage progenitor markers *FOXA2* (also known as hepatocyte nuclear factor 3,beta, HNF3B, P < 0.0001), *SOX17* (P = 0.0003) and *CXCR4* (P < 0.0001) increased significantly at the end of the first stage of induction (compared to control group) and dropped significantly by the end of the second stage, indicating hepatocyte maturation (Fig. 3c). These results were also confirmed by immunofluorescence assay (Fig. 3d,e). To validate the cell function of the induced hepatocytes, we performed the indocyanine green (ICG) assay. Our data clearly show that cells took up ICG post-induction (Fig. 3f), verifying the function of the *in vitro* induced hepatocytes.

### Function of Transplanted hMSGMSCs *in Vivo*

To show that induced cells can function *in vivo*, we transplanted induced chondrocyte pallets subcutaneously under the back skin of severe combined immune deficient (SCID) mice. Chondrocyte-like cells could be seen 2 weeks after transplantation via histological assay. We also observed the expression of glycosaminoglycans and collagen II, which are typical of cartilage, by Alcian blue and immunohistochemical staining, respectively (Supplemental Fig. S1 a-e). These results indicate that the induced pellets have chondrocyte function when transplanted *in vivo*. For osteogenic differentiation, scaffolds seeded with hMSGMSCs were induced in osteogenic media for 14 days *in vitro*, then transplanted subcutaneously into SCID mice. After 12 weeks *in vivo*, while the scaffolds were mostly absorbed, bone matrix was still detected using H&E (Supplemental Fig. S1 f,g). These tests demonstrate that the induced hMSGMSCs can mature and function when transplanted *in vivo*.

Considering the extraordinary hepatic differentiation potential of hMSGMSCs, we explored their differential potential *in vivo*. We used hMSGMSC transplantation to rescue acute liver damage. A 2/3 partial hepatectomy was performed on the SCID mice, and labeled hMSGMSCs were transplanted by tail-intravenous injection immediately after the partial hepatectomy (Fig. 3g). To observe hMSGMSC survival *in vivo*, we euthanized the animals 14 days post-injection and harvested the animal organs for immunofluorescence assays. Labeled cells were detected in the liver, spleen, thymus, heart, lung, pancreas, kidney and skin, and the liver tissue contained the highest abundance of labeled cells. To evaluate the differentiation status of hMSGMSCs after transplantation, we performed a co-immunostaining assay for mature hepatocyte markers. The neonatal and mature hepatocyte markers AFP, KRT18 and ALB were coexpressed in a significant portion of the labeled cells in the liver tissue (Fig. 3h). Next, to test the expansion ability of the detected cells, we isolated labeled hMSGMSCs by flow cytometry sorting and reseeded them into culture *in vitro*. The cells continued to expand and maintained the expression of the marker proteins. These results show that hMSGMSCs do proliferate and mature after they are transplanted into acutely injured liver, suggesting the therapeutic potential of hMSGMSCs in treating diseases.

### Defining the Molecular Characteristics of hMSGMSCs

**Figure 4:**
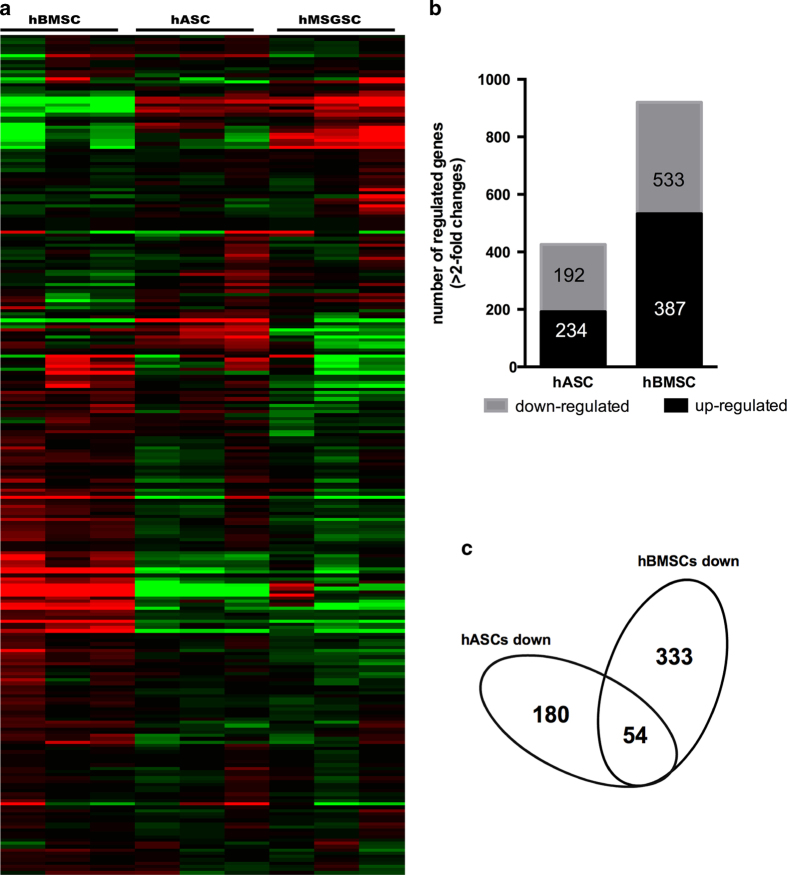
Gene expression profiles by microarray analysis. Clustering was performed to visualize the correlations among the replicates and varying sample conditions. Up- and down-regulated genes are represented in red and green colors, respectively (**a**). Differentially expressed gene numbers of hASCs and hBMSCs compared with hMSGMSCs are shown (**b**). Venn diagram of down-regulated genes compared with hMSGMSCs. The overlapping area represents common differentially expressed genes shared by hASCs and hBMSCs (**c**).

To further investigate the biological characteristics of hMSGMSCs, human bone marrow-derived (hBMSCs) and adipose-derived (hASCs) mesenchymal stem cells, as well as hMSGMSCs, were sent to Phalanx Biotech Group, Inc., for gene expression profile analysis. Differentially expressed genes (DEGs) were identified by P values < 0.05 based on the comparison between the expression levels in hADSCs and hBMSCs with those in hMSGMSCs. In total, 387 up-regulated and 533 down-regulated genes were detected when comparing hBMSCs with hMSGMSCs. Furthermore, 234 up-regulated and 192 down-regulated genes were detected in hASCs compared with hMSGMSCs (Fig. 4b,c). As shown in the clustering analysis based on 257 selected differentially expressed genes (Fig. 4a), hMSGMSCs showed a closer relationship to hASCs than hBMSCs, which was also confirmed by principal component analysis (PCA) (Supplemental Fig.S2). Functional annotations of the 54 common down-regulated genes revealed the enrichment of genes primarily related to the immune response, receptor binding and extracellular regions. We also applied pathway enrichment analysis to the common down-regulated genes and found genes enriched in cytokine-cytokine receptor interactions, the chemokine signaling pathway and the NOD-like receptor signaling pathway (Table 2). Based on the above analyses, we can observe reduced levels of extracellular components and signal transduction in hMSGMSCs.

### Teratoma Assay using hMSGMSCs

To establish whether hMSGMSCs were responsible of forming teratomas, 5 SCID mice were subcutaneously injected with expanded passage 3 hMSGMSCs, with 5 × 10^6^ cells injected per mouse. The mice were examined at 8 weeks post-injection and no tumor formation was observed.

## Discussion

Stem or progenitor cells have been defined in major salivary glands; however, their precise characteristics and functions have not yet been explored. Additionally, limited data are available regarding the presence of mesenchymal stem cells in human minor salivary glands. Our study provides the description of mesenchymal stem cells isolated from human minor salivary glands. In this study, we demonstrate the existence of cells with self-renewal capacity and multi-lineage differentiation potential in human minor salivary glands. We also provide evidence that hMSGMSCs transplanted *in vivo* demonstrate engraftment and proliferation. These findings indicate the potential application of hMSGMSCs in regenerative medicine.

Isolation of adult stem cells from the connective tissue of various sources has been achieved during recent years. It is believed that salivary gland stem/progenitor cells are located at the intercalated ducts of the salivary glands and may differentiate into both acinar and striated/excretory ductal cells^26^,^27^. Approximately 800-1000 minor salivary glands are located in the oral mucosa^13^. These cells are promising candidates for cell therapy because of their easy accessibility and abundant sources for cell isolation. Recently, cells with slow cycling properties have been reported to exist in the basal layer of the excretory ducts in mice minor salivary glands in the mouse soft palate. Some label-retaining cells (LRCs) were observed in the acinar ducts and showed myoepithelial characteristic^19^. With various sources of mesenchymal stem cells be identified with or without prior damage to the tissue^28^, we propose that the mesenchymal tissue between the acinar-ductal epithelial structures may also contain stem cells of mesenchymal origin, similar to other organs such as bone marrow^29^. In our study, we were able to observe proliferative markers and positive stem cell markers expressed on the basal layer of the excretory ducts and in the connective tissue between the acinar-ductal epithelial structures in normal human minor salivary glands without previous injury or radiation.

Upon explant culture of human minor salivary glands, we were able to obtain a population of self-renewing fusiform cells, named hMSGMSCs. Immunophenotypic analysis using flow cytometry revealed that hMSGMSCs show mesenchymal stem cell markers, with strong expression of CD29, CD44, CD73, CD90, CD105 and CD166^30^. Similar to other mesenchymal stem cells, hMSGMSCs did not express CD45 or CD34. However, in contrast to some animal salivary gland studies, CD117 (also known as c-kit) was not detected in hMSGMSCs^31^, which is in accordance with other human studies^32^. hMSGMSCs also represent a cell population with enhanced stem cell characteristics, as shown by the expression of SOX2, SSEA-1 and nestin. SOX2 has been reported to regulate pluripotency as well as adult stem cells and tissue homeostasis in several adult epithelial tissues^33^,^34^. SOX2 also controls the formation of several cell types during fetal development, including the foregut endoderm and the nervous system, which may explain the multi-differentiation potential of hMSGMSCs^35^. The germ-line stem cell marker SSEA-1 has been used for enriching neural stem cells^36^. Additionally, the high expression of nestin supports the potential use of hMSGMSCs in tissue engineering, indicated by a recent study showing that nestin-positive cells are clearly reactivated after tissue injury^37^.

Much work has been performed on the construction of tissues *in vitro* for incurable diseases such as osteoarthritis. Cell therapy via mesenchymal stem cells (MSCs) has shown the most attractive results in both preclinical and clinical research. Consistent with other MSCs, hMSGMSCs showed great capacity for mesodermal differentiation *in vitro,* as indicated by mRNA expression and tissue-specific staining. Furthermore, when hMSGMSCs were transplanted into mice, we were able to observe mature cell types and matrix formation in the induced pellets and cell-seeded scaffolds. With the advantageous of causing less injury at the donor site, hMSGMSCs deserve further investigation for tissue engineering and regenerative medicine for the potential clinical treatment of incurable diseases.

The phenomenon of transdifferentiation has been reported in adult stem cell research^38^. In this study, we also observed the transdifferentiation of hMSGMSCs towards ectodermal neural cells and endodermal hepatic cells. Multiple animal studies have demonstrated the positive impact of cell therapy on liver regeneration^2^. Inspired by a strong differentiation potential towards hepatocytes, we injected undifferentiated hMSGMSCs into an acute liver injury animal model. When the cells were transplanted *in vivo*, we observed the expression of cell survival and mature hepatocyte markers expressed. Though many aspects of hMSGMSC transplantation remain to be explored and improved, the fact that the cells remained engrafted for weeks after transplantation establishes them as promising alternative candidates for *in vivo* modeling and autologous therapy of human diseases.

In summary, we have isolated and characterized a mesenchymal stem cell population with self-renewal and multi-potent differentiation capacity that resides in human minor salivary glands. We have also demonstrated the therapeutic potential of hMSGMSCs by *in vivo* transplantation. These findings highlight the importance of hMSGMSCs in regenerative medicine and autologous cell therapy.

## Methods

### Ethical Statement

The research was carried out in accordance with the Declaration of Helsinki (2013) of the World Medical Association and approved by the Institutional Review Board at Plastic Surgery Hospital, Peking Union Medical College.

### Mice

Female severe combined immune deficiency (SCID/Beige) mice at 6-10 weeks of age were obtained from the Academy of Military Medical Sciences (Beijing, China). The animal experiment protocol was approved by the Animal Care and Use Committee of Plastic Surgery Hospital, Peking Union Medical College. All surgeries were performed under 1% sodium amobarbital anesthesia.

### Isolation and Culture of Stem Cells from Human Minor Salivary Glands (hMSGMSCs)

With the donors’ consent, human minor salivary glands were collected from mucosal tissue discarded during reconstructive surgeries for cleft lip patients. Visible vessels and connective tissue were removed during dissection. The gland tissues were minced into pieces no larger than 0.5 mm^3^ using scissors, and they were then placed on the bottom of a cell culture flask. The flask was left in the culture incubator for 4-6 hours before culture media was added.

The cells were maintained at 37°C and 5% CO_2_. For primary culture, we used DMEM/F12 culture medium supplemented with 10% fetal bovine serum (FBS), 1% Penicillin-Streptomycin (5,000 U/mL) and 1% GlutaMAX™ Supplement (Gibco). The medium was changed every 3 days after day 5. At day 10-14, fibroblast-like cells were isolated by 0.25% trypsin via cloning rings as described^39^. The passaged cells were expanded in Mesenchymal Stem Cell Medium (ScienCell Research Laboratories, Carlsbad, CA). For passaging, the cells were split 1:3 using the trypsin method every 3-5 days.

### Cell Proliferation and Colony Formation Assays

The cells were trypsinized and counted at passage 3. The cells were seeded at a density of 1 × 10^4^ per well in a 96-well plate for the MTT assay. Six replicate wells were made for each sample, and the absorbance at 490 nm was read every day for 7 continuous days. A cell growth curve was depicted, and the doubling time was estimated as described^40^. Two hundred cells were seeded into 100 mm culture dish and cultured for 14 days for the colony formation assay. Colonies were stained with crystal violet^41^. The procedure was repeated 3 times to calculate the colony formation efficiency.

### Flow Cytometry

Cultures at passage 3 were harvested and counted, and 1 × 10^6^ cells were used for each test. For cell surface markers analysis, the cells were incubated with antibodies for 30 minutes at 4 °C, washed twice with staining buffer (BD Biosciences, San Jose, CA) and resuspended in 1% paraformaldehyde (PFA) before the test. For intracellular antibody staining, single-cell suspensions were fixed in 4%PFA in PBS for 30 minutes, washed and permeabilized in 0.2% Triton X-100 for 10 minutes, and then washed and incubated with the antibodies. Each sample with 1 × 10^4^ cells were generated by FACSAria^TM^ II (BD) and analyzed using FlowJo X 10.0.7r2(Tree Star Inc., OR).

### Immunohistochemistry and Immunofluorescence Assay

All frozen sections and cell samples were fixed in ice-cold methanol and acetone for 10 minutes. The tissue sections were stained with hematoxylin and eosin (H&E). For the immunofluorescence staining assay, sections or cells were permeabilized with 0.2% Triton X-100 for 10 minutes and blocked in 10% FBS for 1 hour at room temperature. Primary antibodies were incubated at 4 °C overnight and then washed with PBS supplemented with 1% BSA. Secondary antibodies were incubated for 30 minutes at room temperature. The cells were mounted with DAPI and imaged with a Nikon TE2000-S microscope.

### RNA Isolation, Microarray, RT-PCR, and qPCR

Total RNAs were extracted from cells using the RNeasy Mini kit (QIAGEN) according to manufacture’s instructions and quantified using a NanoDrop instrument. The RNA was reverse transcribed by M-MLV (Invitrogen) according to the manufacture’s instructions. Real-time quantitative PCR (qPCR) was run on a Light-Cycler Roche480 (Roche Molecular Systems, Pleasanton, CA). The fold changes were normalized to the levels of GAPDH. For microarray assays, to determine the RNA integrity, the RIN values were ascertained using an Agilent RNA 6000 Nano assay. Microarray analysis was performed by the Phalanx Biotech Group using the Human HOA5.1 OneArray (Phalanx Biotech Group, San Diego, CA).

### Multilineage Differentiation

#### Mesodermal Differentiation

hMSGMSCs at passage 3 were subjected to osteogenic, chondrogenic, and adipogenic differentiation regimens^8^ and were analyzed for the mRNA expression of lineage-specific markers by RT-PCR. Special staining methods using Alizarin S Red, Alcian blue and Oil Red O were applied to visualize mineralization, acidic glycans and lipid droplets, respectively.

#### Neurogenic Differentiation

hMSGMSCs at passage 3 were pretreated with DMEM supplemented with 20% FBS for 24 hours, then changed into pre-induction media supplemented with 10 ng/mL bFGF for 16 hours. Next, induction media supplemented with 200 μM BHA was added, and the cells were fixed or collected for further assays after 5 hours.

#### Hepatic Differentiation

A three-stage induction protocol was used to induce hepatocytes. First, the hMSGMSCs were pretreated with media supplemented with 20 ng/mL EGF and 10 ng/mL bFGF for 2 days. Then, the induction media supplemented with 20 ng/mL HGF, 10 ng/mL bFGF and 4.9 mM nicotinamide was added and changed every other day for 7 days. Finally, a second-stage induction medium supplemented with 20 ng/mL oncostatin M, 1 μM dexamethasone and ITS premix was added and changed every other day for 7-14 days. The cells were then fixed or collected for further assays.

### Animal Models and hMSGMSCs Transplantation

A two thirds partial hepatectomy was performed on the mice to establish an acute liver injury model^42^. hMSGMSCs at passage 3 labeled with CM-Dil (Invitrogen) following the manufacturer’s instructions were suspended in 150 μL PBS and injected via the tail vein immediately after hepatectomy. Tissues were collected after two weeks for immunohistochemistry assays.

### Statistical Analysis

Two-tailed paired t tests were used to investigate the changes in gene expression after induction. One-way ANOVA tests were used to analyze gene expression change during hepatocyte induction and Fisher’s LSD tests were used as post-hoc tests for multiple comparison. One-way ANOVA tests were also used to analyze the flow cytometry data to define the effect of passaging effect *in vitro*. Each experiment was performed in triplicate. P-values < 0.05 were considered statistically significant.

## Additional Information

**How to cite this article**: Lu, L. *et al*. Characterization of a Self-renewing and Multi-potent Cell Population Isolated from Human Minor Salivary Glands. *Sci. Rep.* **5**, 10106; doi: 10.1038/srep10106 (2015).

## Supplementary Material

Supplementary Information

## Figures and Tables

**Table 1:** Flow cytometry analysis of human minor salivary gland mesenchymal stem cells.

	Average	s.d.
Mesenchymal stem cell markers		
CD29	99.91%	0.14%
CD44	99.81%	0.16%
CD73	93.83%	2.99%
CD90	94.46%	3.04%
CD105	76.80%	5.95%
CD166	97.93%	2.18%
CD80	2.02%	2.57%
CD86	0.56%	0.03%
HLA-ABC	99.27%	0.58%
HLA-DR	0.40%	0.20%

Embryonic stem cell markers		
Sox2	68.23%	9.67%
Oct4	1.53%	1.06%
Nanog	18.33%	5.75%
SSEA-1	27.60%	1.83%
Nestin	98.24%	1.55%

Other markers		
CD34	0.20%	0.00%
CD45	0.17%	0.17%
CD49f	1.20%	0.00%
CD338	0.00%	0.00%

Averaged percentage of positive cells for the cell markers are shown in the table. Each marker was tested for passage 3 cells derived from three different samples. Flow cytometry analysis for passaged cells at passage 5,10,15,20 are shown in Supplementary Table S1. s.d.: standard deviation.

**Table 2:** The enriched GO functions and KEGG pathways for common up-regulated between BMSCs and ASCs compared with hMSGMSCs.

Category	Term	Gene number	P Value
GOTERM_MF	G-protein-coupled receptor binding	10	1.15E-09
GOTERM_MF	cytokine activity	11	1.12E-08
GOTERM_BP	inflammatory response	12	1.72E-07
GOTERM_BP	response to wounding	14	4.76E-07
GOTERM_BP	response to external stimulus	17	1.71E-06
GOTERM_MF	receptor binding	16	4.63E-06
GOTERM_BP	behavior	12	6.26E-06
GOTERM_CC	extracellular space	14	6.50E-06
GOTERM_BP	immune response	14	8.84E-06
GOTERM_CC	extracellular region part	16	1.20E-05
GOTERM_BP	response to chemical stimulus	18	3.09E-05
GOTERM_CC	extracellular region	22	7.76E-05
GOTERM_BP	immune system process	15	1.01E-04
GOTERM_BP	signal transduction	26	2.81E-04
GOTERM_BP	JAK-STAT cascade	4	6.90E-04
GOTERM_BP	response to stimulus	27	0.00294
GOTERM_MF	glycosaminoglycan binding	5	0.00324
GOTERM_BP	cytokine-mediated signaling pathway	4	0.00375
GOTERM_BP	response to lipopolysaccharide	4	0.00490
GOTERM_BP	response to stress	16	0.00625
GOTERM_BP	response to biotic stimulus	7	0.00718
GOTERM_BP	response to other organism	6	0.00941
KEGG_PATHWAY	chemokine signaling pathway	11	1.58E-07
KEGG_PATHWAY	cytokine-cytokine receptor interaction	10	2.89E-05
KEGG_PATHWAY	NOD-like receptor signaling pathway	6	4.18E-05

The differentially expressed genes in hBMSCs and hASCs were grouped in categories classified by P value <0.05 compared with hMSGMSCs. BP: biological processes; CC: cell components; MF: molecular functions.
