# Supplementary material for: Characterization of a Self-renewing and Multi-potent Cell Population Isolated from Human Minor Salivary Glands
Source: Sci Rep. 2015 Jun 9;5:10106. doi: 10.1038/srep10106 (PMC4460572; doi:10.1038/srep10106)
Supplement: Supplementary Information [file srep10106-s1.pdf]

Article Title: Characterization of a Self-renewal and Multi-potent Cell Population Isolated from Human Minor Salivary Glands

Authors:

Lin Lu[1,#] E-mail address: lulinjade@gmail.com

Yan Li[1,3#] E-mail address: s2007148@126.com

Ming-juan Du[4] E-mail address: s2007150@126.com

Chen Zhang[5] E-mail address: nightglow123@gmail.com

Xiang-yu Zhang[6] E-mail address: nchot@163.com

Hai-zhou Tong[6] E-mail address: 838472562@qq.com

Lei Liu[6] E-mail address: ssydll@126.com

Ting-lu Han[6] E-mail address: 1634877010@qq.com

Wan-di Li[6] E-mail address: wandili@hotmail.com

Li Yan[1] E-mail address: yanli102894@gmail.com

Ning-bei Yin[6] E-mail address: ningbeiyin@gmail.com

Hai-dong Li[6] E-mail address: 93041992@qq.com

Zhen-min Zhao[1,2,*] Tel: (86)010-88772233; Fax:(86)010-88964379;

E-mail address: zhaozhenmin0098@163.com

# These two authors contributed equally to this work

* Corresponding author

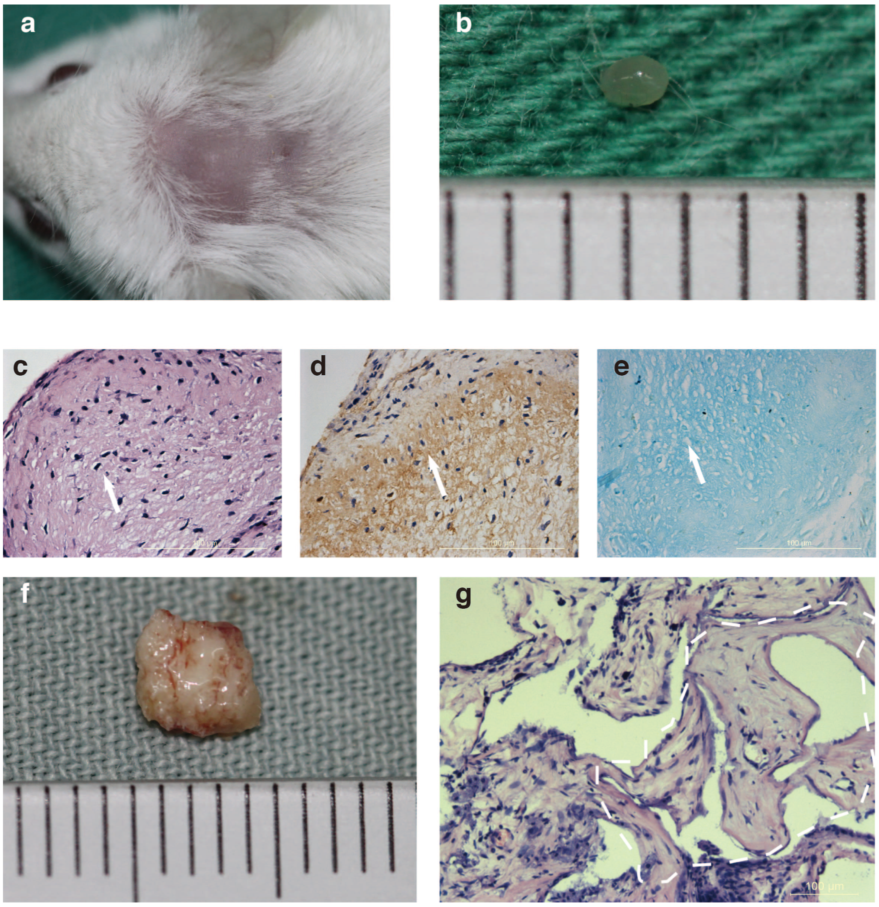

Supplemental Figure S1. ***In vivo* differentiation of hMSGSCs.** Subcutaneously transplanted chondrogenic pellet (a) and pellet harvested after two weeks *in vivo* (b). H&E (c), collagen II immunohistology (d) and Alician blue staining (e) of chondrogenic pellet harvested two weeks after transplanted *in vivo*. Scaffold harvested after transplanted *in vivo* for 12 weeks (f). H&E visualization for bone formation of transplanted scaffold, bone matrix formed shown in white frame (g).

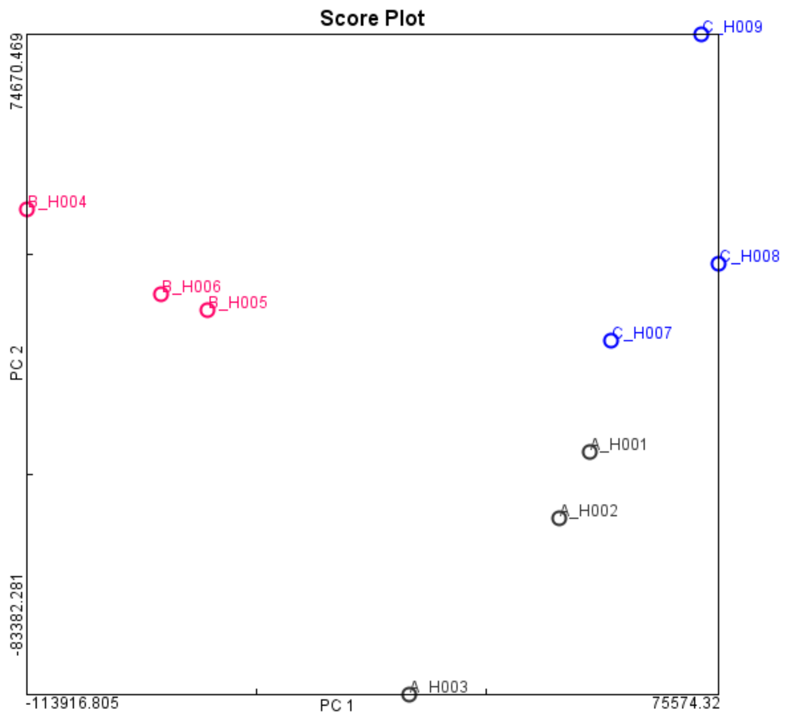

Supplemental Figure S2. PCA plot. The variable of the first three principal components (PC1, PC2, PC3) for this study are 38.2%, 16.7% and 14.7%, respectively.

Markers	Passage 5	Passage 10	Passage 15	Passage 20
CD29	99.99±0.02%	98.60±1.3%	98.10±0.67%	97.50±0.29%
CD44	99.90±0.10%	99.53±0.20%	99.52±0.42%	98.50±0.70%
CD73	99.80±0.12%	98.63±1.32%	99.37±0.24%	99.67±0.24%
CD90*	91.70±2.95%	82.40±8.05%	62.90±4.90%	60.65±5.65%
CD105*	67.24±6.27%	68.82±8.96%	54.67±2.91%	36.27± 1.90%
SSEA-1***	7.99±1.21%	3.98±0.17%	1.81±0.14%	0.92±0.04%

Supplementary Table S1: Flow cytometry analysis for passaged cells at passage 5,10,15,20. Averaged percentage of positive cells for the cell markers are shown in the table. Each marker was tested cells derived from three different samples. Results are offered in average ± s.e.m.. One-way ANOVA was done for each cell marker. No significance was detected among CD29, CD44 or CD73. Significance difference was shown among CD90(p=0.031), CD105(p=0.013) and SSEA-1(p=0.0002). *p<0.05, **p<0.01, ***p<0.001.
